# Catecholamines production by kidney tissue and mesangial cell culture is differentially modulated by diabetes

**DOI:** 10.1590/2175-8239-JBN-2020-0236

**Published:** 2021-05-31

**Authors:** Roseli Peres Moreira, Nadia S. C. Bertoncello, Juliana Almada Colucci, Danielle Yuri Arita, Maria Claudina Camargo de Andrade, Fernanda Aparecida Ronchi, Tatiana Sousa Cunha, Dulce Elena Casarini

**Affiliations:** 1 Universidade Federal de São Paulo, Departamento de Medicina, Disciplina de Nefrologia, São Paulo, SP, Brasil.; 2 Universidade Federal de São Paulo, Instituto de Ciência e Tecnologia, São Paulo, SP, Brasil.

**Keywords:** Diabetes Mellitus, Catecholamines, Norepinephrine, Epinephrine, Dopamine, Diabetes Mellitus, Catecolaminas, Noradrenalina, Epinefrina, Dopamina

## Abstract

**Introduction:**

According to the International Diabetes Federation, the number of people with diabetes mellitus may reach 700 million in 2045. Catecholamines are involved in the regulation of several kidney functions. This study investigates the effects of hyperglycemia on catecholamines' metabolism in kidney tissue from control, diabetic, and insulin-treated diabetic rats, both *in vivo* and *in vitro*.

**Methods:**

Male Wistar-Hannover rats were randomized into: control, diabetic, and insulin-treated diabetic groups. Diabetes was induced by a single injection of streptozotocin, and diabetic treated group also received insulin. After 60 days, blood and kidney tissue from all groups were collected for catecholamines' quantification and mesangial cells culture.

**Results:**

diabetic rats had lower body weight, hyperglycemia, and increase water intake and diuresis. Additionally, diabetes promoted a sharp decrease in creatinine clearance compared to control group. Regarding the whole kidney extracts, both diabetic groups (treated and non-treated) had significant reduction in norepinephrine concentration. In mesangial cell culture, catecholamines' concentration were lower in the culture medium than in the intracellular compartment for all groups. Norepinephrine, epinephrine, and dopamine medium levels were increased in the diabetic group.

**Conclusion:**

The major finding of the present study was that 8 weeks of diabetes induction altered the kidney catecholaminergic system in a very specific manner, once the production of catecholamines in the excised kidney tissue from diabetic rats was differentially modulated as compared with the production and secretion by cultured mesangial cells.

## Resumo

**Introdução:**

Segundo a Federação Internacional de Diabetes, o número de pessoas com diabetes mellitus pode chegar a 700 milhões em 2045. As catecolaminas estão envolvidas na regulação de várias funções renais. Este estudo investiga os efeitos da hiperglicemia no metabolismo das catecolaminas no tecido renal de ratos controle, diabéticos e diabéticos tratados com insulina, tanto in vivo como in vitro.

**Métodos:**

Os ratos Wistar-Hannover machos foram randomizados em: grupos controle, diabéticos e diabéticos tratados com insulina. O diabetes foi induzido por uma única injeção de estreptozotocina, e o grupo diabético tratado também recebeu insulina. Após 60 dias, sangue e tecido renal dos grupos foram coletados para quantificação de catecolaminas e cultura de células mesangiais.

**Resultados:**

ratos diabéticos apresentaram peso corporal mais baixo, hiperglicemia, e aumento da ingestão de água e diurese. Ademais, o diabetes promoveu uma redução acentuada na depuração de creatinina comparado com o grupo controle. Quanto aos extratos de rim total, ambos os grupos diabéticos (tratados/não tratados) tiveram redução significativa na concentração de noradrenalina. Na cultura de células mesangiais, a concentração de catecolaminas foi menor no meio de cultura do que no compartimento intracelular para todos os grupos. Níveis médios de noradrenalina, adrenalina e dopamina estavam aumentados no grupo diabético.

**Conclusão:**

O principal achado deste estudo foi que 8 semanas de indução de diabetes alteraram o sistema catecolaminérgico renal de maneira muito específica, já que a produção de catecolaminas no tecido renal excisado de ratos diabéticos foi modulada diferencialmente comparada com produção e secreção por células mesangiais cultivadas.

## Introduction

According to the International Diabetes Federation, the number of people with diabetes mellitus (DM) may reach 700 million in 2045^1^. This number is alarming since diabetes influences quality of life and life expectancy of patients. Multiple efforts have been made in the search for a better understanding of the disease and most importantly its complications. If not kept under a tight control, the hyperglycemia leads to numerous chronic macro- and microvascular diseases including stroke, neuropathy, retinopathy, and kidney disease^2^. Among these, the most detrimental complication is diabetic kidney disease, representing a major concern of public health worldwide, irrespective of the type of diabetes^3^.

Diabetic kidney disease is a leading cause of mortality and morbidity in patients with diabetes and consists of glomerular sclerosis and fibrosis caused by the metabolic and hemodynamic changes associated with diabetes. After a long period of 'silence' with normal urinary albumin excretion rate, the first clinical sign of the disease appears^3^. The presence of persistent pathologic albuminuria greater than 300mg/24hrs (macroalbuminuria) accompanied by abnormally elevated plasma creatinine or diminished glomerular filtration rate (GFR) determine diabetic kidney disease^4^. Histologically, diabetic kidney disease manifests as diffuse or nodular mesangial expansion, tubular and glomerular basement membrane thickening, as well as interstitial fibrosis^5^.

Mesangial cells are major constituents of the renal glomerulus. They play a pivotal role in the regulation of GFR and participate in the development of functional and morphological glomerular abnormalities^6^. Mesangial cells correspond to one third of the decapsulated glomerular cell population and allow multiple physiological roles in normal glomerular function such as synthesis and secretion of the extracellular matrix. Additionally, they provide structure and stability to the filtration barrier and, moreover, they are the target of a wide variety of hormones and growth factors^7^. This cell type constitutes a potential site for catecholamines production, expressing all synthesizing and degrading enzymes necessary to the production cascade^8^.

Catecholamines are well-known hormones that play an important role in the regulation of a variety of kidney physiological functions, such as sodium and water metabolism and arterial pressure control, and a consequent causative role in several common diseases including hypertension, dyslipidemia (metabolic syndrome), and diabetes^9^. In several experimental models, DM is induced by streptozotocin (STZ) that selectively destroys pancreatic islet β-cells mimetizing human type 1 DM, resulting in insulin deficiency, hyperglycemia, polydipsia, and polyuria^10^. This type of experimental DM is accompanied by an increase of catecholamines in the brain, heart, and pancreas 11 ^,^ 12. In the kidney, Marco *et al*. (2008)^6^ showed that DM induced changes on catecholamines in primary mesangial cell culture from the non-obese diabetic mouse. The authors observed an increase of dopamine and norepinephrine production/secretion in mesangial cell culture and kidney tissue, which could contribute to the impairment of kidney function. The authors described that diabetes alters catecholamines' production by interfering with both synthesizing and degrading enzymes, suggesting a possible role of catecholamines in the pathogenesis of acute and chronic kidney complications of DM^6^^.^

Therefore the purpose of this study was to investigate the profile of catecholamines in whole kidney tissue and also in primary mesangial cell culture obtained from control and STZ-treated Wistar rats that could contribute for diabetic kidney disease development.

## Material and Methods

### Ethics statement

All experimental procedures followed Institutional Guidelines for Care and Use of Laboratory Animals, and the protocols were approved by the Ethics Committee of Universidade Federal de São Paulo, Brazil (CEP/UNIFESP - 0240/08).

### Experimental design

The current study was carried out to investigate the effects of hyperglycemia on catecholamines in kidney tissue from STZ-diabetic and insulin treated STZ-diabetic rats, both *in vivo* and *in vitro*. Male Wistar-Hannover rats received a single STZ injection to make them diabetic and were accompanied during 60 days after the onset of diabetes. Urine and blood samples were collected and kidney tissue was removed. Kidneys were used for morphological analysis, tissue catecholamines' quantification, and mesangial cell culture. Catecholamines' concentration was determined in mesangial cell culture from control (CT), STZ-diabetic (D) and insulin-treated STZ-diabetic rats (TD).

### Animals and experimental groups

Two-month-old male Wistar-Hannover rats (n=8/group) obtained from Centro de Desenvolvimento de Modelos Experimentais para Medicina e Biologia of Universidade Federal de São Paulo (CEDEME), were used throughout this study, and randomly assigned into 3 groups: control (CT), diabetic (D), and insulin-treated diabetic (TD). Animals were fed standard laboratory chow and given tap water *ad libitum* while housed (4-5 *per* cage) in a temperature and humidity-controlled room (22°C and 60±5%), with a 12:12 h light-dark cycle (lights on at 7 a.m.) for 60 days.

Diabetes was induced by a single tail vein injection of STZ after a 12 h fasting (STZ, 60 mg/kg body weight; Sigma, Chemical, St. Louis, MO; in freshly prepared 0.01 M citrate buffer, pH 4.5^13^. Age-matched control animals were injected with citrate buffer only. STZ-injected animals were allowed to drink 5% glucose solution overnight to prevent initial drug-induced hypoglycemic mortality. Following this period, animals were kept for 3 days with free access to food and water. STZ-injected animals exhibited massive glycosuria and hyperglycemia within a few days, and diabetes was confirmed by measuring fasting blood glucose concentration 4 days after drug injection. Fasting blood glucose was determined in blood samples obtained by tail prick, using a strip operated glucometer (Accu-Check^®^ Sensor, Roche, Switzerland), and rats with a fasting plasma glucose greater than 250 mg/dL were considered diabetic. TD rats started receiving 2 UI (daily) of Neutral Protamine Hagedorn insulin (NPH Humulin^®^ N, Eli Lilly Laboratory and Company, Brazil)^13^ after 4 days of STZ injection.

### Metabolic cages and sample collection

At week 8 of the experimental protocol, rats were housed in individual metabolic cages. After 1 day of adaptation, water and food intake, as well as 24 h urine volume were measured. Urine was collected and centrifuged at 3,000 rpm, glucose, pH, and density were determined qualitatively using commercially available strips, and their levels were graded based on details provided by the manufacturer (Labor Strips-URS10^®^ Urine Reagent Strips 10, TECO Diagnostics, USA)^14^.

At the end of the experimental period, animals were anesthetized intramuscularly with ketamine hydrochloride (100 mg/kg)/ xylazine (12 mg/kg). Blood samples and left kidneys were collected and stored at -80°C until the day of creatinine and catecholamines evaluation. Right kidneys was removed for primary mesangial cell culture.

### Glomerular filtration rate

Urine total protein (BioRad Protein Assay Reagent, BioRad Labs, Hercules, CA) was measured in urine aliquots collected as described above^15^. GFR was estimated by creatinine clearance. Creatinine was measured in urine and plasma samples by the Jaffe method using Creatinina K^®^ kit (Labtest Diagnostica, Brazil)^16^.

### Primary culture of mesangial cells

Mesangial cells (MC) were obtained by outgrowth from rat glomeruli, isolated by differential sieving, as previously described by our group^17^^,^ 18. Briefly, glomeruli were isolated from the kidney using a graded-sieving technique with stainless steel and nylon meshes under sterile conditions. Primary cultures of mesangial cells from CT, D, and TD rats were established by plating isolated glomeruli (300 glomeruli/cm) in Dubelcco's Modified Eagle Medium (DMEM, Invitrogen, Carlsbad, CA)^19^ containing 20% fetal calf serum (FCS), penicillin (50 U/mL), HEPES (15 mM), glutamine (2 mM), and D-glucose (5 mM). Cells were allowed to grow at 37°C in 5% CO_2_ and 95% air in cell culture flasks. The culture medium was replaced every 36 h. After 3 weeks, cells were harvested with trypsin and the subcultures were grown in the same culture medium. Cells were used between the third and fifth subculture and characterized by classic methods using the following criteria: morphological appearance of stellate cells, immunofluorescence staining of the extracellular matrix for type IV collagen and fibronectin, negative immunofluorescence staining for human factor VIII antigens (glomerular endothelial cells) and cytokeratin (parietal epithelial cells), and positive immunofluorescence staining for actin and myosin.

At subconfluence, mesangial cell cultures from all groups were plated and maintained in the culture medium indicated above. After 72 h, cells were rinsed twice with PBS and culture media was replaced with a medium without FBS, to keep the cells in the G0 phase of the cell cycle. The culture medium was then collected over the last 24 h and stored at -80°C until use. Cells were rinsed with PBS, lysed with 1 mM Tris-HCl buffer, pH 7.5, and stored at -80°C until use. The catecholamines' concentration in the cell lysate and culture medium was determined by HPLC, as described below.

### Catecholamines quantification

Whole kidneys were homogenized in 15 mL 0.1 M perchloric acid, Na_2_S_2_O_5_ (sodium metabisulfite), and 10 µL internal standard dihydroxybenzylamine (DHBA) (1 mM). The homogenates were kept refrigerated overnight and centrifuged at 12,000 rpm for 50 minutes. Catecholamines were extracted from the culture medium (2 mL) and cell lysates (0.8 mL) using Al_2_O_3_ (alumina) and DHBA. Catecholamines from kidney tissue and mesangial cell cultures (culture medium and cell lysate) were measured using ion-pair reverse phase chromatography coupled with electrochemical detection (0.5 v) as described by Di Marco et al^8^. Fast isocratic separation was obtained using an RP 18 Aquapore cation F micron, Brownlee Column (Applied Biosystems, San Jose, CA) (4.6x250 mm) eluted with the following mobile phase: 0.02 M sodium dibasic phosphate, 0.02 M citric acid, pH 2.64, 10% methanol, 0.12 mM Na_2_EDTA and 566 mg/L heptanesulfonic acid. The total sample analysis lasted 30 min. Catecholamines' levels found in cell homogenates as well as in the correspondent culture medium were normalized by intracellular protein content.

### Statistical analysis

Results are expressed as means ± SEM. Statistical differences were determined by one-way analysis of variance (ANOVA). Differences were considered significant at P<0.05 comparing groups CT, D, and TD.

## Results

### *In Vivo* Study

Table 1 shows that D animals presented a reduction in body weight compared to CT (266.90 *vs* 317.50 g) and TD (266.90 *vs* 298.00 g) groups. Blood glucose in D reached levels as high as 3 times those of CT (371.40 *vs* 113.40 mg/dL) group and the insulin treatment was successful in bringing blood glucose back to the normal range (371.40 *vs* 114.50 mg/dL).

**Table 1:** Physiologic parameters of control(CT), diabetes (D), and treated diabetes (TD)

	CT	D	TD
**Body weight (g)**	317.50 ± 2.00	266.90 ± 2.70^*^	298.00 ± 1.10^#^
**Blood glucose (mg/dL)**	113.40 ± 0.60	371.40 ± 11.40^*^	114.50 ± 1.00^#^

Values are mean ± SEM.

* p<0.05 vs CT;

# p<0.05 vs D; n=6.

We used metabolic cages to assess different physiologic and metabolic parameters such as water intake and urine output (Table 2). D animals had a 3.5 times increase in water intake when compared to CT (109.30 *vs* 31.30 mL/day) and 1.6 times compared to TD group (109.30 *vs* 66.50 mL/day). Regarding urine excretion, D rats showed a significantly higher diuresis when compared to C (90.00 *vs* 14.70 mL/day), which was partially reversed in the TD group (90.00 *vs* 44.50 mL/day).

**Table 2:** Physiologic parameters of control (CT), diabetes (D), and treated diabetes (TD) groups from metabolic cages

	CT	D	TD
**24 hours water intake (mL/day)**	31.30 ± 1.80	109.30 ± 16.00^*^	66.50 ± 6.70^*^
**24 hours urine output (mL/day)**	14.70 ± 1.50	90.00 ± 12.40 ^*^	44.50 ± 6.90^* #^
**Urine total protein (mg/24hs)**	13.60 ± 1.00	27.90 ± 3.00^*^	19.50 ± 3.10
**Creatinine clearance (mL/min)**	2.20 ± 0.30	0.80 ± 0.10^*^	2.10± 0.20^#^
**Urine pH**	7.90 ± 0.20	6.80 ± 0.20^*^	6.90 ± 0.20^*^
**Urine glucose (mg/dL)**	Negative	> 1000	< 600

Values are mean ± SEM.

* p<0.05 vs CT;

# p<0.05 vs D; n=6.

Proteinuria and creatinine clearance were also affected by diabetes. A significant increase in proteinuria was observed in both diabetics groups, D and TD (27.9 ± 3.00 and 19.50 ± 3.10 mg/24hs respectively) compared to CT group (13.6 ± 1.00 mg/24hs). For the creatinine clearance, while CT and TD groups showed normal values (2.20 and 2.07 mL/min respectively), the D group had a sharp decrease in this parameter (0.80 mL/min). The urinary protein / creatinine ratio (rP/C), used to assess injury to the glomeruli when there is still no clinical evidence, demonstrated that group D had this parameter approximately twice as high as for groups CT and TD (rP/C: 1.61; 2.95 and 1.70 for CT, D and TD respectively). Both D and TD also showed decrease in urinary pH (CT= 7.90; D=6.80; TD=6.90) and increased urinary glucose (CT=negative; D>1000; TD<600 mg/dL) when compared to the control group (Table 2).

**Figure 1:**
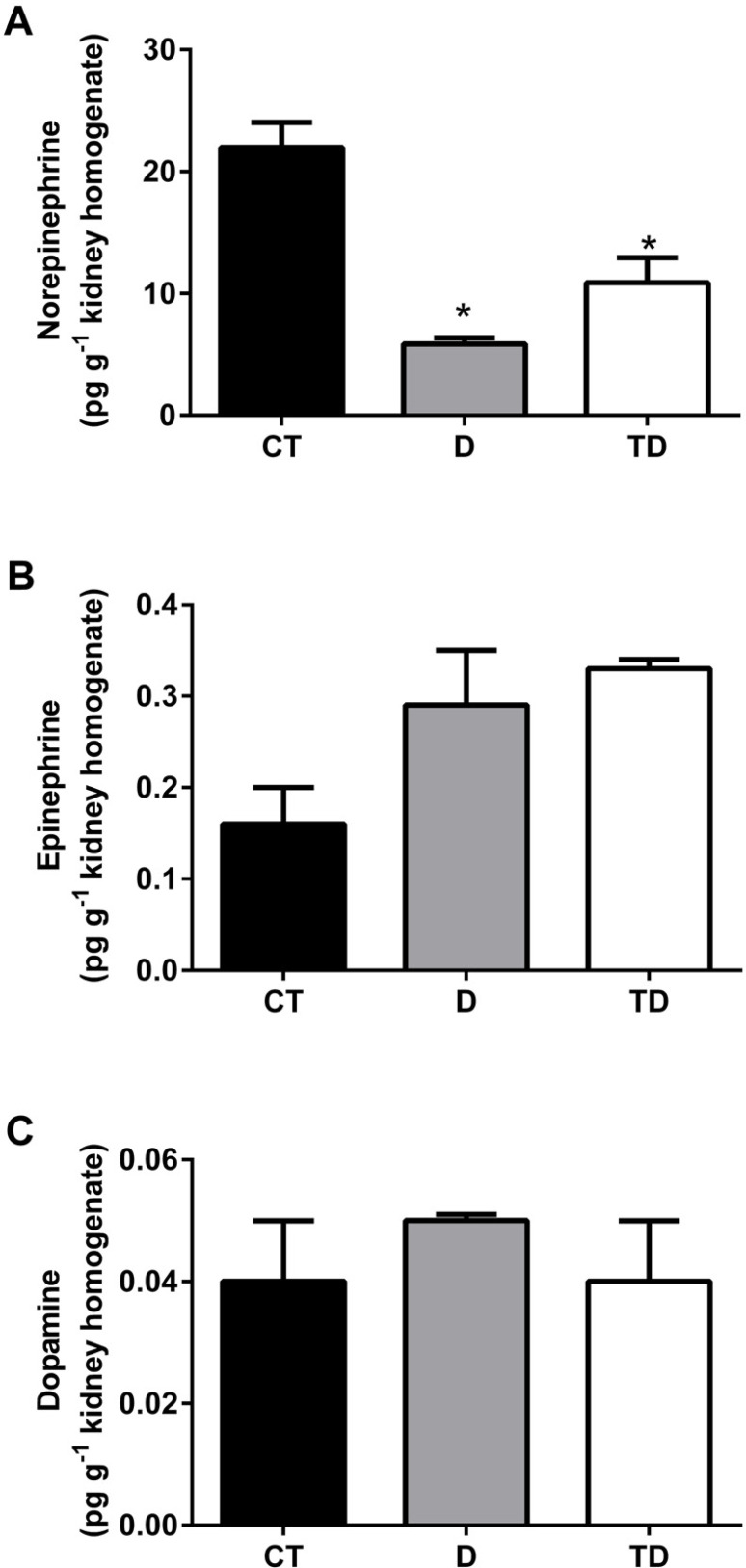
Catecholamines' quantification in the whole kidney extracts from Wistar rats. Norepinephrine (1A), epinephrine (1B), and dopamine (1C). All values are means ± SEM, n = 6, *p<0.05 vs CT. Groups: CT - control; D - diabetic; TD - treated diabetic.

Figure 1 shows the catecholamines' content in whole kidney extracts. Both D and TD groups had a significant decrease in norepinephrine levels (CT=21.98+2.06; D=5.86+0.6*; TD=10.88+2.06* pg·g^-1^ kidney homogenate, *p*<0.05) as seen in Figure 1A. Renal epinephrine (CT=0.16+0.04; D=0.29+0.06; TD=0.33+0.01 pg·g^-1^ kidney homogenate) and dopamine concentrations (CT=0.04+0.01; D=0.05+0.001; TD=0.04+0.01 pg·g^-1^ kidney homogenate) were not different among the groups.

### Mesangial cell culture

Catecholamines' levels were significantly lower in the culture medium than in the intracellular compartment in three groups (CT, D and TD). Diabetes increased the concentration of norepinephrine, epinephrine, and dopamine in the culture medium from D group (43.50+5.70; 149.00+33.60 and 57.90+11.20 pg·mg^-1^ cellular protein, respectively, *p*<0.05) compared to CT (18.20+1.30; 13.30+1.90 and 7.20+0.90 pg·mg^-1^cellular protein, respectively, *p*<0.05) and TD (26.90+2.80; 17.60+2.3 and 6.80+0.70 pg·mg^-1^ cellular protein, respectively, *p*<0.05) groups.

**Figure 2:**
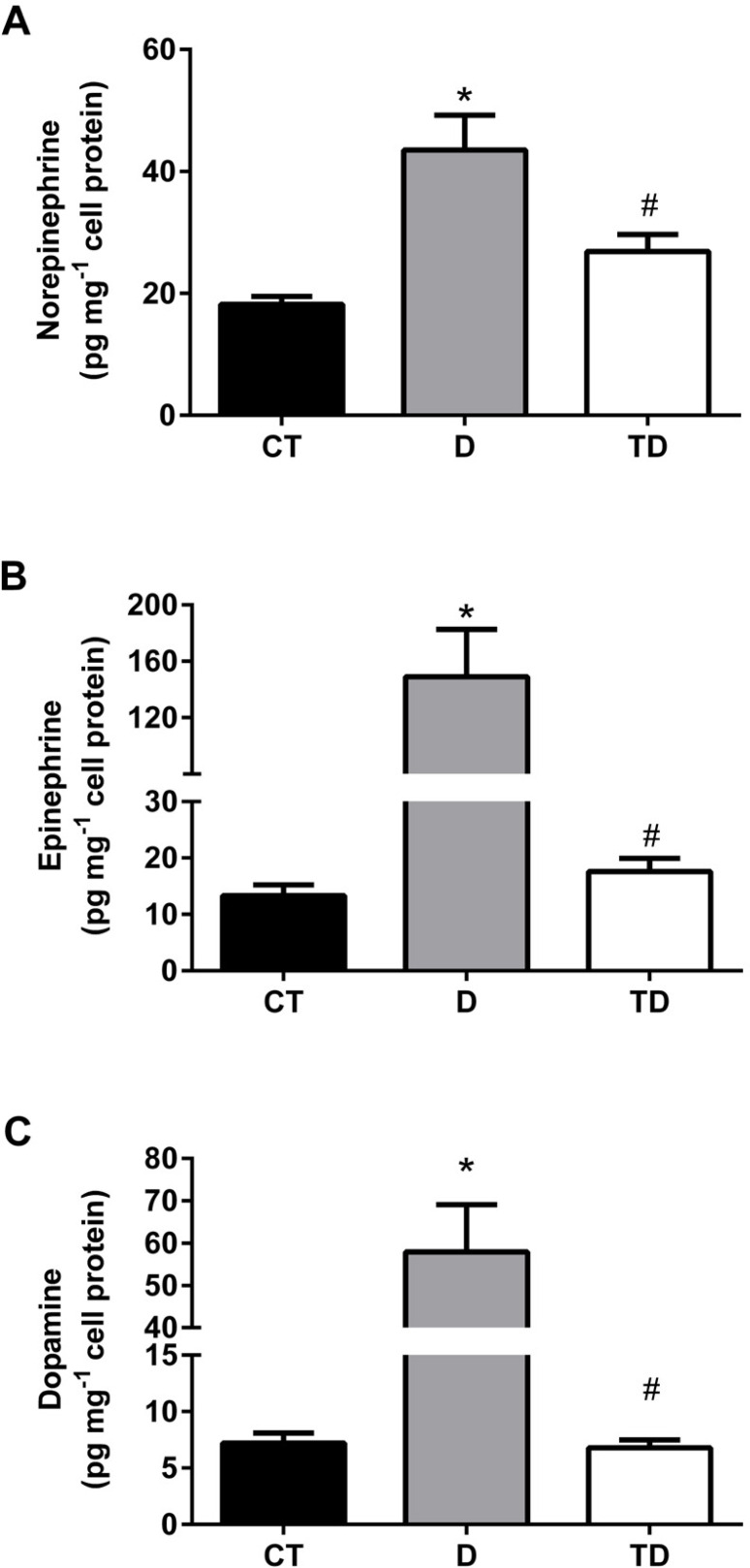
Catecholamines' quantification in the extracelular compartment of MC (medium) from Wistar rats. Norepinephrine (2A), epinephrine (2B), and dopamine (2C). All values are mean ± SEM, n = 6, *p<0.05 vs CT; #*p*<0.05 vs D.

**Figure 3:**
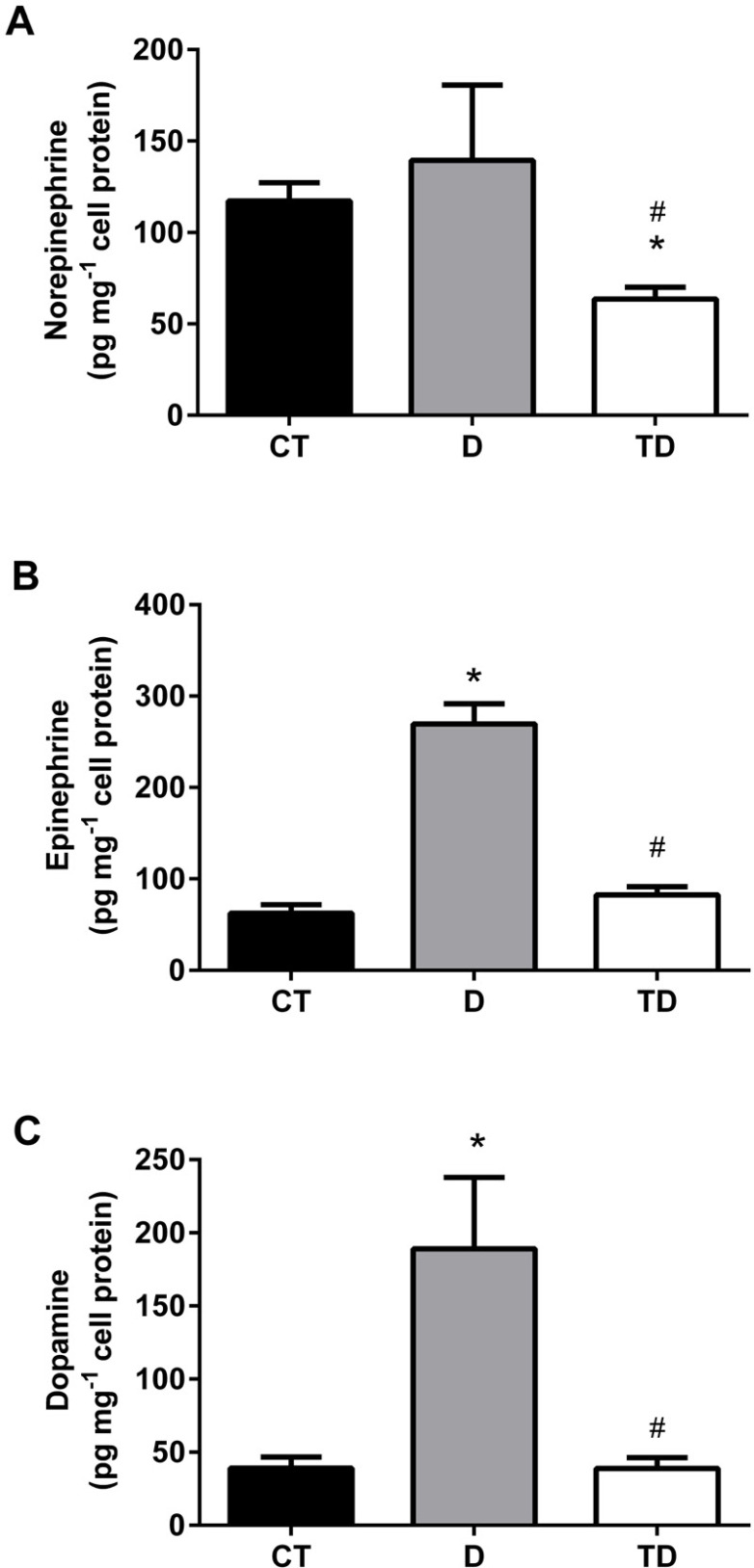
Catecholamines' quantification in the intracellular compartment of primary MC (lysate) from Wistar rats. Norepinephrine (3A), epinephrine (3B), and Dopamine (3C). All values are mean ± SEM, n = 6, *p<0.05 vs CT; #*p*<0.05 vs D. Groups: CT - control; D - diabetic; TD - treated diabetic.

In the cell lysate, epinephrine and dopamine were increased in D (269.60+21.9 and 189.10+48.70 pg·mg^-1^ cellular protein, respectively, *p*<0.05) compared with CT (62.60+9.30 and 39.10+7.60 pg·mg^-1^ cellular protein, respectively, *p*<0.05) and TD (82.50+9.00 and 38.80+7.50 pg·mg^-1^ cellular protein, respectively, *p*<0.05) groups. On the other hand, the intracellular content of norepinephrine was not different among D (139.40+41.10), CT (117.30+9.90), and TD groups (63.50+6.50·g^-1^ cellular protein, *p*<0.05) as showed in Figures 2 and 3.

## Discussion

The major finding of the present study was that 8 weeks of diabetes altered kidney catecholaminergic system in a very specific manner, once the production of catecholamines by excised kidney tissue from STZ-diabetic rats is differentially modulated as compared with the production and secretion by cultured mesangial cells.

Even though the STZ-diabetic rat develops characteristic lesions similar to those found in human diabetic kidney disease, this animal model does not develop hypertension, making it a good model for evaluating the effects of hyperglycemia itself. In fact, in a previous study from our group using the same animal model and treatment protocol, Ronchi et al. have shown that STZ rats treated or not with insulin, do not present increased blood pressure^13^. This adaptation was also previously observed, and in some cases, authors describe a decrease in blood pressure^20^. The absence of hypertension in this model would allow us to focus on metabolic effects of diabetes on kidney function and catecholamines production, independently of the effects of systemic hypertension.

In this animal model we observed marked differences in urinary biomarkers of diabetic kidney disease, such as an increase in total protein excretion and an important decrease in creatinine clearance. It is important to mention that STZ-animals exhibit progressive proteinuria and an early decrease in kidney function. On the other hand, they present histological changes, including glomeruli and the tubulointerstitial lesions, only after 3-6 months of diabetes. This is in accordance with a previous study from our group, using the same animal model, where Ronchi et al. (2007)^13^ did not find any structural alteration analyzing kidney histology of diabetic animals during the early development of the disease^21^^,^ 22. This is also supported by other studies, showing that STZ-animals can be resistant to the development of histological kidney damage^23^. However, it is important to mention that structural pathological changes associated with diabetic kidney disease, including the deposition of extracellular matrix, glomerular basement membrane thickening, activation of proliferative pathways, tubular atrophy, and mild to moderate albuminuria, have been described in STZ animals 6 months after the induction on diabetes^24^^,^ 25.

The STZ animal model is useful for elucidating the mechanism associated with the early pathogenesis of diabetic kidney disease^25^ considering that it only takes as little as a few days for hyperglycemia to start upregulating the pathways that lead to this complication^26^. In this study, the alteration of glomerular filtration of diabetic animals associated with polyuria characterizes the initial stage of diabetic kidney disease^13^^,^ 27.

Several lines of evidence have suggested that an early state of imbalance between nitric oxide (NO) pathway and renin-angiotensin system (RAS)^28^ leading to an enhancement in the peripheral sympathetic nervous system activity is implicated in the pathogenesis of kidney dysfunctions accompanying DM^27^^,^ 29 ^-^ 31. In addition, angiotensin II as the main effector of RAS and a powerful vasoconstrictor, leads to the release of catecholamines from the adrenal medulla and prejunctional nerve endings, predisposing to hypertension associated with DM^31^. Di Marco et al. (2008)^6^ used mesangial cells from non-obese diabetic mice and their results suggested an implication of diabetes on both synthesizing and degrading catecholamines' enzymes, suggesting a possible role of these components in the pathogenesis of acute and chronic kidney complications of DM.

In fact, circulating norepinephrine is increased in people with poorly controlled diabetes or hypoglycemia^32^. On the other hand, it has been reported that the levels of plasma catecholamines are decreased^32^^,^ 33, or unchanged^11^^,^ 34 in response to diabetes. Although information exists, the validity of inferences about the activity of the sympathetic nervous system on diabetes based only on the circulating plasma catecholamines is questionable since plasma levels of the amines do not necessarily represent tissue-specific sympathetic activity^35^.

Apart from controversies regarding catecholamines' circulating levels in response to diabetes, there are also discrepancies regarding tissue catecholamines concentration that seem to depend on the animal model, severity, and duration of diabetes^32^^,^ 36. In a previous study, our group demonstrated that whole kidney homogenates from non-obese diabetic (NOD) mice present increased production and secretion of norepinephrine as compared to normoglycemic NOD and Swiss mice^6^. However, Fushimi et al. (1988)^37^ have reported that as the duration of the diabetic state lengthens in rats, there is a time-proportional stepwise decrease in plasma catecholamine response^36^. Giachetti (1978)^38^ also described, using the db/db mice, that depending on the duration of diabetes, kidney norepinephrine concentration is not influenced or is decreased in response to diabetes^38^, and this type of alteration was also observed in cardiac tissue from diabetic rats^39^^,^ 40. This is similar to the clinical course observed in human diabetics, which also includes a reduction of catecholamine excretion after the appearance of autonomic neuropathy. In diabetic patients with autonomic neuropathy, plasma norepinephrine concentration, used as an index of sympathetic nervous activity, is low. This decrease is, however, only found in patients with a long duration of diabetes with clinically severe autonomic neuropathy^36^.

In response to insulin-induced hypoglycemia in diabetic patients with autonomic neuropathy, the plasma levels of epinephrine and norepinephrine were significantly lower than in diabetics without autonomic neuropathy and healthy individuals. These results demonstrated that responses to insulin-induced hypoglycemia of epinephrine and norepinephrine are affected in diabetics with autonomic neuropathy^41^. Moreover, changes in the sympathetic nervous system resulting from complications of DM lead to different clinical manifestations, such as postural hypotension and changes in various visceral functions. It has been reported that autonomic neuropathy is related to increased mortality in type 1 and type 2 DM and that patients with advanced diabetic autonomic neuropathy have decreased plasma norepinephrine^36^. As described by Granados et al. (2000)^43^, norepinephrine basal levels are diminished in patients under cold responses and orthostatic tests with definite and severe autonomic neuropathy, evidencing a response modulated by stress. In that same study, epinephrine levels in diabetic patients with and without autonomic neuropathy were not different, suggesting that adrenal function is not altered. These authors found no association of blood glucose or glycated hemoglobin levels with the severity of neuropathy^43^.

In the present study, it was not possible to determine whether the decrease on kidney norepinephrine concentration was a consequence of neuropathy or a protective response to kidney damage, considering that increased sensitivity to norepinephrine has been described in diabetic kidneys^44^. Still regarding the possible protective response related to reduced concentration of norepinephrine in kidney tissue, as a consequence of sympathetic activation, norepinephrine raises glucose levels worsening animal metabolic state^45^. It is important to mention that the marked decrease on the concentration of catecholamines in kidney tissue could also be a consequence of kidney hypertrophy, not accompanied by a proportional increase in total norepinephrine content; thus, the concentration of amine (nanograms per gram) is decreased^38^. A closer analysis of the data concerning kidney norepinephrine, epinephrine, and dopamine concentrations show that diabetes does not induce a proportional decrease in the three catecholamines, suggesting that the important decrease in norepinephrine concentration is probably related to changes in peripheral adrenergic nerve function, which seems to correlate with the severity of the experimental diabetic syndrome, as observed in previous studies^38^.

Luippold et al. (2004)^46^ reported that chronic kidney denervation prior to the induction of STZ-induced DM possessed a considerable renoprotective action against glomerular hyperﬁltration^46^. These ﬁndings support the view that adrenergic components are directly related to the impact on kidney and hemodynamic functions in uncontrolled DM. In the clinical context, Feyz et al. (2020)^47^ studied the effect of kidney sympathetic denervation on 60 hypertensive patients. The study concluded that despite significant reduction in blood pressure, denervation did not promote significant change in neither catecholamines nor RAS components. However, to date, the responsible mediators as well as underlying mechanisms involved in kidney sympathetic denervation are not completely understood.

Our group and others have demonstrated that primary MC have the necessary biosynthetic machinery to produce catecholamines *in vitro*, suggesting that they can act as a paracrine/ autocrine hormone system, contributing to the regulation of glomerular hemodynamic and kidney microcirculation^8^^,^ 48. Considering the importance of MC in the maintenance of glomerular function, that catecholamines play a causative role in diabetic kidney disease^49^, and that underlying mechanisms involved in this pathology are not completely understood, our group has also demonstrated that MC isolated from NOD mice (type I DM) produced and secreted higher levels of dopamine and norepinephrine by increasing the activity of the tyrosine hydroxylase and inhibiting the monoamine oxidase activity 6.

Herein, we described that cultured MC isolated from STZ rats (cell lysate), produced higher norepinephrine, epinephrine, and dopamine levels as compared to control, and insulin treatment was capable to reduce them. On the other hand, MC from STZ diabetic rats secreted increased amounts of epinephrine and dopamine, but not of norepinephrine (culture medium), and this imbalance was corrected by insulin, demonstrating the important role of hyperglycemia on the development of these alterations. It is well known that MC control the glomerular function due to their ability to contract and reduce the glomerular capillary surface area, and synthesize several hormones. Local generation of these vasoactive substances may profoundly alter kidney function, independent of systemic hemodynamic modiﬁcations^6^^,^ 50. In the normal state, tonic contraction by MC counteracts intraglomerular hypertension to maintain GFR constant by autoregulation. Evidence for the involvement of MC in DM was ﬁrst presented by Kimmelsteil and co-workers^51^, who showed that the mesangium was the initial glomerular site associated with diabetic nephropathy. Subsequent studies have shown that in the diabetic state, hyperﬁltration would result if MC could not isometrically and isotonically contract to counteract intraglomerular pressure. Thus, evidence from a variety of diabetic models suggests that dysfunctional MC are partially responsible for the condition of diabetic hyperﬁltration. However, it is likely that not one, but several signaling pathways are involved in the pathological response of MC in diabetes.

Our results show a marked increase of dopamine concentration and secretion by cultured MC, and this study design allowed us to show for the first time that, in response to diabetes, dopamine production by kidney tissue extract is differentially modulated as compared with the production by MC from diabetic rats. This difference suggests a possible protective role of dopamine in MC, in response to the progression of kidney disease in STZ animal model. In fact, it has been described that increased intrarenal dopamine levels inhibited hyperfiltration, decreased markers of oxidative stress, and inhibited macrophage infiltration, whereas decreased intrarenal dopamine production had the opposite effect. Indeed, Marwaha et al. (2004)^52^ showed that STZ-induced diabetic rats present reduced kidney dopamine D1 receptor function, which in turn is due to a decrease in dopamine D1 receptor expression and a defect in the coupling of the receptor to the G protein. Authors describe that this abnormality in D1 receptor expression along with defective receptor G protein coupling and function is partially caused by hyperglycemia and may contribute to sodium retention seen in type I diabetes. In the literature it was described that dopamine play a pivotal role in diabetic nephropathy^49^.

In summary, we showed that STZ administration produced a signiﬁcant increase in blood glucose level and reduction of body weight, along with other typical symptoms of diabetes, including polydipsia, polyphagia, and polyuria, which were partially reversed by insulin treatment. With the onset of diabetes, it was observed a decrease in creatinine clearance, indicating the successful establishment of a diabetic kidney disease model. Also, results showed that 8 weeks of diabetes altered the kidney catecholaminergic system in a very specific manner, once the production of catecholamines by excised kidney tissue from diabetic rats is differentially modulated as compared with the production and secretion by cultured MC. The regulation of this hormonal cascade and its potential contribution to the intrarenal control of glomerular function in diabetes are not completely understood. However, with the genomic and proteomic tools becoming increasingly available, several transcription factors and regulators are being identified regarding this pathway, which could permit a greater understanding of the contribution that changes in cellular and tissue catecholamines represent in the pathogenesis of diabetic kidney disease. Moreover, a better understanding of the pathophysiology of kidney disease associated with diabetes will aid the development of new and complementary drug targets. Finally, we would also like to stress the importance of the MC model, since it provides a convenient model for the study of kidney synthesis and release of catecholamines lacking neuronal contribution.
